# Clinical monitoring of peripheral perfusion: perspective on ProCess

**DOI:** 10.1186/s13054-014-0619-5

**Published:** 2014-10-30

**Authors:** John PR Moore, John F Fraser

**Affiliations:** Department of Intensive Care, Nambour Hospital, Nambour, Queensland 4560 Australia; Critical Care Research Group, The University of Queensland and The Prince Charles Hospital, Brisbane, Queensland 4032 Australia

We read with interest the commentary by Lima and Bakker [1]. The authors note that there are two phases in the shock state that differ in the degree of uncoupling of macrocirculatory and microcirculatory function. They suggest that a shift to flow-based resuscitation may offer benefit for the critically ill by prioritising microcirculatory perfusion. This commentary is of particular interest when viewed in the context of ProCess [2], the recent negative early goal-directed therapy study.

The absence of a treatment effect in this trial from the therapies one would normally expect to resuscitate the microcirculation warrants consideration. Resuscitation attempts that reverse microcirculatory dysfunction early in the shock state appear to offer clinical benefit [3]. Conversely, in the phase of established shock the primary function of the microcirculation may be limiting damage by preventing spread of infection [4] and harmful excess oxygen delivery in parallel with mitochondrial shutdown and reduced oxygen consumption [5]. Microcirculatory dysfunction in established organ failure may therefore in fact be adaptive. Re-recruitment as a result of zealous resuscitation could hence be predicted to result in detrimental tissue effects. The ProCess trial utilised aggressive resuscitation strategies, without an endpoint that reflected microcirculatory perfusion in the context of ongoing metabolic need. This could be predicted to result in successful resuscitation of some patients and harm to others, dependent on their individual phase of illness.

We offer caution that despite the negative results from the ProCess trial, the concept of early goal-directed therapy should not yet be discounted, but should be revisited once valid clinical measures of microcirculatory and metabolic function are available.
